# Association between left ventricular remodeling and lipid profiles in obese children: an observational study

**DOI:** 10.3389/fped.2024.1308887

**Published:** 2024-02-23

**Authors:** Ying Tang, Guang-bin Yang, Jun Chen, Ye Chen, Li-chun Hua

**Affiliations:** ^1^ Department of Ultrasound, Children’s Hospital of Nanjing Medical University, Nanjing, Jiangsu, China; ^2^ Department of Ultrasound, The Fourth Affiliated Hospital of Nanjing Medical University, Nanjing, Jiangsu, China

**Keywords:** childhood obesity, ventricle, echocardiography, cardiac function, dyslipidemia

## Abstract

**Objective:**

Childhood obesity has become a prominent issue in the society, which can lead to left ventricular remodeling and severe cardiovascular complications in adulthood. It is beneficial to identify the causes of left ventricular remodeling so that targeted measures can be taken to prevent the cardiovascular disease. Therefore, this study aimed to explore the relationship between left ventricular remodeling and changes in blood lipid indexes in obese children.

**Methods:**

This study was conducted on 40 healthy non-obese children and 140 obese children diagnosed in the pediatric health department of our hospital. Clinical data collected from the two groups were compared. Echocardiography was performed to examine left ventricular configuration and cardiac function. Multiple linear regression analysis was conducted to assess the independent effects of blood lipid levels on echocardiographic parameters. Blood lipid indicators among different left ventricular structural patterns which were classified according to left ventricular mass indexes and relative wall thickness were compared.

**Results:**

Obese children exhibited significantly increased height, weight, body mass index (BMI), body fat percentage (BFP), blood pressure, triglycerides, total cholesterol, left ventricular internal diameter (LVIDd), interventricular septum (IVSd), left ventricular posterior wall diastolic thickness (LVPWd), myocardial mass (LVM) and relative wall thickness (RWT), as well as lower high-density lipoprotein cholesterol (HDL-C) and left ventricular ejection fraction (LVEF) compared to the non-obese children (*P *< 0.05). Multiple linear correlation analysis showed LVM had a significantly positive correlation with BMI (*r* = 3.21, *P* = 0.002) and SBP (*r* = 2.61, *P* = 0.01); LVMI had a significantly negative correlation with HDL-C (*r* = −2.45, *P* = 0.015); RWT had a significantly positive correlation with SBP (*r* = 2.50, *P* = 0.013) but a significantly negative correlation with HDL-C (*r* = −2.35, *P* = 0.02). Furthermore, there were significant differences in HDL-C values among children with different ventricular configurations (*P *< 0.05), with the lowest HDL-C value recorded in the concentric hypertrophy group.

**Conclusion:**

Obese children will develop left ventricular remodeling. The left ventricular configuration indexes are most significantly associated with serum HDL-C. Lower HDL-C level contributes to severer left ventricular hypertrophy, indicating a concentric hypertrophy pattern.

## Introduction

Childhood obesity has emerged as one of the most challenging public health issues in China. Within the European region of the World Health Organization, approximately one-third of children are classified as overweight or obese (1). With the potential consequences it poses to physical well-being, childhood obesity has received sufficient attention from society. Moreover, it leads to severe cardiovascular complications in adulthood and serves as a significant risk factor for coronary artery disease and congestive heart failure, ultimately raising rates of morbidity and mortality (2).

Childhood obesity can lead to thickening and enlargement of the walls of the heart's ventricles (3). This physiological adaptation is aimed at supporting the increased workload on the heart (4). Moreover, obesity has been found to be associated with the accumulation of fat and inflammation in the heart, which may have detrimental effects on ventricular function (5). Additionally, childhood obesity is linked to lipid disorders, which affect the levels of various blood lipids (6). The alterations in blood lipid profile, including changes in triglycerides (TG), total cholesterol (TC), and high-density lipoprotein cholesterol (HDL-C) and low-density lipoprotein cholesterol (LDL-C), are common metabolic abnormalities associated with obesity (7). These changes are known to potentially induce structural modifications in the left ventricle, leading to the development of left ventricular hypertrophy (LVH) in obese children. As a consequence, the overall health of children is at higher danger due to the subsequent functional impairments (2). However, extensive studies on the association between lipid levels and left ventricular mass and geometry in children are lacking.

This study adopted body mass index (BMI) combined with body fat percentage (BFP) as the screening criteria for obesity. We utilized two-dimensional echocardiography and tissue Doppler imaging to evaluate the left ventricular structural changes in obese children compared to normal children. Additionally, we examined the relationship between these changes and blood lipid markers. The aim of this study was to accurately analyze the relationship of childhood obesity with cardiac structural abnormalities, as well as with the alterations in the blood lipid profile. Furthermore, we sought to assess the risk of early cardiac changes and emphasize the importance of primary prevention of cardiovascular events in obese children and strengthening of early intervention and preventive measures.

## Materials and methods

### Study design

This study was conducted with 140 outpatient obese children included in Children's Hospital of Nanjing Medical University from March 2018 to September 2022. Additionally, a control group of 48 healthy children who underwent physical examinations (with BMI below the 85th percentile) were included in the study. Obesity was defined as a BMI at or above the 95th percentile for children and teenagers of the same age and sex according to the National Child Measurement Programme (8). BMI is calculated by dividing a person's weight in kilograms by the square of its height in meters. The study followed the principles of the Declaration of Helsinki and was approved by the hospital ethics committee. Written informed consent was obtained from the parents and the subjects.

### Participants

The inclusion criteria of the obese children were (1) BFP greater than 25% for boys and BFP greater than 30% for girls, (2) children who had normal physical activity (7–8 h per week), which was assessed through surveys of the patients and their parents. The exclusion criteria were (1) children had cardiovascular-related diseases or other chronic diseases, (2) those who had a history of diabetes or treatment with antihypertensive medications, and (3) those who did not cooperate with the inspection.

The inclusion criteria of the healthy children were (1) BFP less than 25% for boys and BFP less than 30% for girls, (2) children with normal echocardiographic examination results. The exclusion criteria were (1) children who had any illness, (2) those who had a history of cardiac arrhythmias or genetic diseases, (3) those who take any medications, and (4) those who did not cooperate with the inspection.

### Data collection

The BMI, BFP, blood pressure values [systolic blood pressure (SBP), diastolic blood pressure (DBP)], fasting blood glucose, fasting insulin level and blood biochemical indicators including TC, TG, HDL-C and TG/HDL ratio were recorded and summarized for obese children.

### BFP measurement

BFP was measured using the INbody 770 body composition analyser. To be specific, after emptying the urine, the participants took a sitting position to rest for 0.5 h, and then stood calmly for 5 min. Next, their shoes and socks were removed before standing relaxed on the test platform with the feet and hands connected to the electrodes. Notably, the thumbs and palms should be in contact with the electrodes (during the test, saline can be applied to the soles and palms to increase the electrical conductivity of the skin), and the arms should be slightly separated from the torso. Finally, the switch of the bioimpedance tester was turned on, and the participants were kept in a quiet position for 3–5 min.

### Blood sample analysis

All participants had their blood collected on an empty stomach. Beckman AU680 automatic biochemistry analyser was used for glucose testing and Roche E411 chemiluminescence detector for the detection of serum insulin level. The blood biochemical parameters including TC, TG, HDL-C and TG/HDL ratio were measured using a Hitachi 7,600 biochemistry instrument.

### Echocardiography

**Figure 1:**
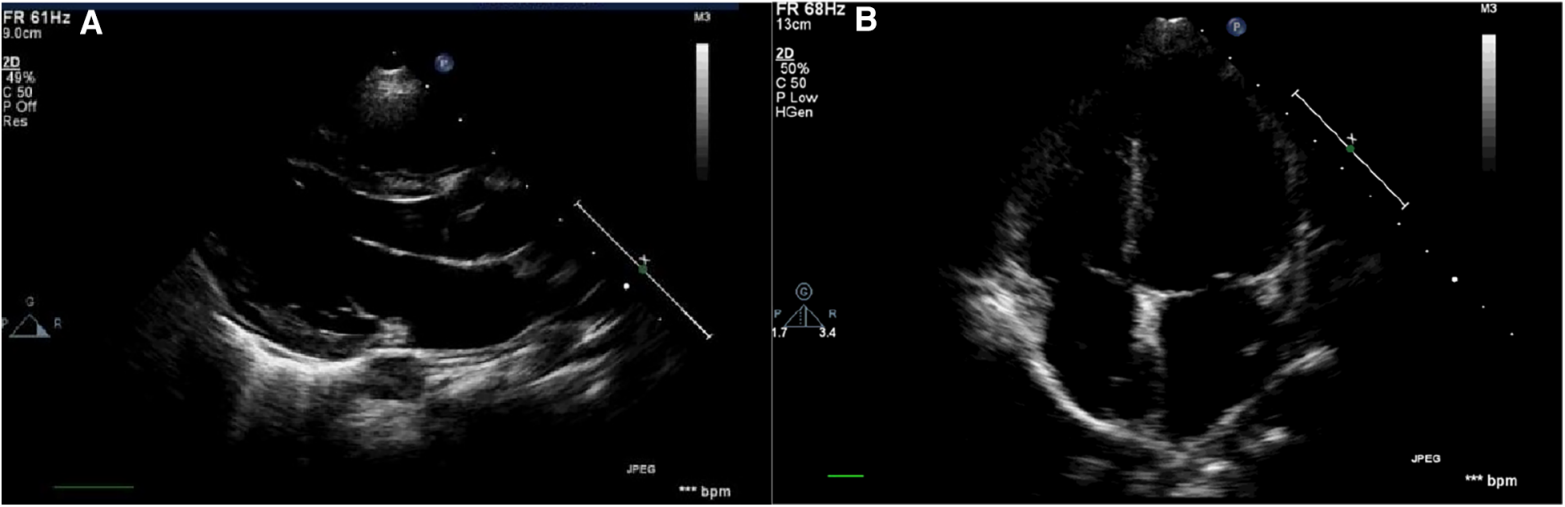
Schematic diagram of the echocardiography. (**A**) Parasternal long-axis view: measurement of left ventricular structural indices including LVIDd, IVSd, LVPWd, as well as left ventricular systolic function indices such as LVEF and LVFS; (**B**) apical four-chamber view of the echocardiogram: measurement of blood flow and tissue Doppler indices associated with mitral and tricuspid valves.

Philips IE33 color Doppler ultrasound diagnostic instrument was used for echocardiography in this study. The S5-1 or S8-3 probe was selected based on the age and transmissibility of the children. The patients were placed in a supine or left lateral position and kept quiet. Then the electrocardiograms were connected, and the probe was placed at the cardiac apex to obtain and store images of the four-chamber, three-chamber, and two-chamber views during three consecutive cardiac cycles when the rhythm was stable (Figures 1A,B).

### Echocardiography data measurements

The ultrasound measurements and recordings were mainly divided into two categories: left ventricular structural indicators including left ventricular end-diastolic dimension (LVIDd), end-diastolic interventricular septal thickness (IVSd), left ventricular end-diastolic posterior wall thickness (LVPWd), and aortic root dimension (AOD); and functional indicators including left ventricular ejection fraction (LVEF) and left ventricular fractional shortening (LVFS). Left ventricular mass (LVM) was calculated as LVM = 0.8 × 1.04 × [(LVIDd + LVPWd + IVSd)3—(LVIDd)3] + 0.6 (g), left ventricular mass index (LVMI) as LVMI = LVM/H2.7 (g/m2.7), and relative wall thickness (RWT) as RWT = 2LVPWd/LVIDd (cm) (9).

Based on the different values of LVMI and RWT, the left ventricle was classified into four types of configurations: normal configuration (normal RWT and LVMI), concentric remodeling (increased RWT but normal LVMI), concentric hypertrophy (increased RWT and LVMI), and eccentric hypertrophy (normal RWT but increased LVMI). LVMI ≥ the 95th percentile was considered increased, with LVMI ≥ 41 g/m^2.7^ for boys and ≥36 g/m^2.7^ for girls, and RWT ≥ 0.41 cm was considered increased (10).

All ultrasound examinations were performed by the same sonographer to avoid measurement errors between different examiners. The examiners were unaware of the participants’ blood pressure, blood biochemical indicators, and other basic information.

### Statistical analysis

The statistical analysis was performed using SPSS 25.0. Continuous variables were expressed as mean ± standard deviation (SD). Normality test and homogeneity of variance test were conducted. Independent sample *t*-tests were employed to compare continuous variables between two groups, while categorical variables used the chi-square test. Comparison of blood lipid indicators among different left ventricular structural pattern groups was conducted using one-way analysis of variance (ANOVA). Furthermore, simple and multiple linear regression analyses were conducted to assess the independent effects of blood lipid levels on echocardiographic parameters. Statistical tests were performed as two-tailed tests, with *P *< 0.05 indicating statistical significance.

## Results

### Clinical characteristics and heart ultrasonic parameters of the obese and non-obese children

A total of 188 children were enrolled in this study, among which 140 children (33 girls and 107 boys, with the mean age 10 years) were obese children and 48 ones were non-obese. The clinical characteristics and heart ultrasonic parameters of the obese and non-obese children are presented in Table 1. The obese group had increased height (151 cm vs. 140 cm), weight (69 kg vs. 29 kg), BMI (29 kg/m^2^ vs. 18 kg/m^2^), BFP (41% vs. 29%), SBP (127 mmHg vs*.* 100 mmHg), DBP (74 mmHg vs. 63 mmHg), TG (1.59 mmol/L vs. 1.37 mmol/L), TC (4.08 mmol/L vs. 3.66 mmol/L), LVIDd (45 mm vs. 36 mm), IVSd (9 mm vs. 6 mm), LVPWd (8 mm vs. 6 mm), LVM (127 g vs. 62 g) and RWT (0.39 cm vs. 0.32 cm), as well as decreased LVEF (65% vs. 67%) and HDL-C (1.16 mmol/L vs. 1.28 mmol/L) compared to the healthy control group (*P* < 0.05). However, no significant differences were observed in terms of gender, age, heart rate, fasting blood glucose, fasting insulin level, TG/HDL ratio and LVMI (*P *> 0.05).

**Table 1:** Comparison of clinical characteristics and heart ultrasonic parameters between the two groups.

Variables	Obese (*n* = 140)	Non-obese (*n* = 48)	*t/X^2^*	*P* value
Gender, boys/girls (*n*)	107/33	31/17	1.51	0.14
Age (years)	10.39 ± 2.09	9.44 ± 3.50	1.52	0.14
Height (cm)	151.13 ± 14.23	140.97 ± 22.37	2.53	0.016
Weight (kg)	68.63 ± 19.36	29.40 ± 14.55	12.13	<0.01
BMI (kg/m^2^)	29.43 ± 4.68	17.66 ± 4.78	13.52	<0.01
Body fat percentage (%)	41.41 ± 6.52	28.81 ± 6.43	11.60	<0.01
Heart rate (bpm)	94.19 ± 19.12	91.81 ± 17.32	0.76	0.45
Systolic blood pressure (mmHg)	127.19 ± 17.20	100.43 ± 14.50	8.68	<0.01
Diastolic blood pressure (mmHg)	73.57 ± 11.82	62.92 ± 11.92	4.86	<0.01
Fasting blood glucose (mmol/L)	4.46 ± 0.41	4.39 ± 0.31	0.97	0.34
Fasting insulin level (μIU/L)	15.14 ± 14.10	11.58 ± 7.92	1.36	0.177
Triglycerides (mmol/l)	1.59 ± 0.80	1.37 ± 0.58	2.09	0.039
Total cholesterol (mmol/L)	4.08 ± 0.82	3.66 ± 1.04	2.81	<0.01
HDL-C (mmol/l)	1.16 ± 0.22	1.28 ± 0.31	3.06	<0.01
TG/HDL ratio	1.48 ± 0.95	1.17 ± 0.86	1.96	0.051
LVIDd (mm)	44.56 ± 4.06	36.17 ± 7.77	7.15	<0.01
IVSd (mm)	8.95 ± 1.88	6.32 ± 1.55	8.72	<0.01
LVPWd (mm)	8.31 ± 1.95	5.78 ± 1.67	8.05	<0.01
LVEF (%)	64.78 ± 10.63	67.23 ± 4.64	1.54	0.125
LVM (g)	127.34 ± 61.04	61.81 ± 35.89	7.03	<0.01
LVMI (g/m^2^)	40.95 ± 12.70	37.79 ± 13.58	1.41	0.16
RWT (cm)	0.39 ± 0.07	0.32 ± 0.05	6.10	<0.01

Independent sample *t*-tests were employed to compare continuous variables between two groups, while categorical variables used the chi-square test. BMI, body mass index; BFP, body fat percentage; TG, triglycerides; HDL-C, high-density lipoprotein cholesterol; LVIDd, left ventricular internal diameter; IVSd, interventricular septum; LVPWd, left ventricular posterior wall diastolic thickness; LVEF, left ventricular ejection fraction; LVM, left ventricular myocardial mass; LVMI, left ventricular mass index; RWT, relative wall thickness.

### Correlation between left ventricular myocardial structural parameters and blood lipids, body mass index, and body fat percentage in obese children

Linear regression analysis was performed to examine the relationship between LVMI, RWT, and variables such as BMI, BFP (BF%), lipid profile parameters (TG, TC, and HDL-C), fasting insulin, fasting blood glucose, and SBP. The results are presented in Tables 2, 3. The simple linear analysis showed that LVM had a significantly positive correlation with BMI (*r* = 0.292, *P* < 0.01), SBP (*r* = 0.287, *P* < 0.01) and DBP (*r* = 0.195, *P* < 0.05), as well as a significantly negative correlation with HDL-C (*r* = −0.22, *P* < 0.01); LVMI was significantly positively correlated with BFP (*r* = 0.185, *P* < 0.05) and TG/HDL ratio (*r* = 0.189, *P* < 0.05), but negatively correlated with HDL-C (*r* = −0.285, *P* < 0.01); RWT had a significantly positive correlation with SBP (*r* = 0.232, *P* < 0.01) as well as a significantly negative correlation with HDL-C (*r* = −0.195, *P* < 0.05) (Table 2). Further multiple linear analysis showed that LVM had a significantly positive correlation with BMI (*r* = 3.21, *P* = 0.002) and SBP (*r* = 2.61, *P* = 0.01); LVMI displayed a significantly negative correlation with HDL-C (*r* = −2.45, *P* = 0.015); RWT exhibited a significantly positive correlation with SBP (*r* = 2.50, *P* = 0.013) but a significantly negative correlation with HDL-C (*r* = −2.35, *P* = 0.02) (Table 3).

**Table 2:** Simple linear regression analysis of correlation between blood lipid, BMI, blood glucose, insulin, systolic blood pressure (SBP), body fat percentage, and parameters related to ventricular morphology.

Parameters	LVM		LVMI		RWT	
	*r*	*P* value	*r*	*P* value	*r*	*P* value
BMI	0.292	<0.01	0.158	0.062	0.085	0.321
BFP	0.036	0.673	0.185	<0.05	0.089	0.297
Systolic blood pressure (mmHg)	0.287	<0.01	0.143	0.092	0.232	<0.01
Diastolic blood pressure(mmHg)	0.195	<0.05	0.104	0.22	0.115	0.18
Fasting blood glucose	0.061	0.479	−0.008	0.928	0.053	0.534
Fasting insulin level	0.094	0.274	0.066	0.444	−0.006	0.949
TG (mmol/L)	0.117	0.168	0.148	0.082	0.133	0.118
Total cholesterol (mmol/L)	0.046	0.588	0.015	0.861	0.045	0.594
HDL-C (mmol/L)	−0.22	<0.01	−0.285	<0.01	−0.195	<0.05
TG/HDL ratio	0.154	0.069	0.189	<0.05	0.126	0.137

BMI, body mass index; BFP, body fat percentage; TG, triglycerides; HDL-C, high-density lipoprotein cholesterol; LVM, left ventricular myocardial mass; LVMI, left ventricular mass index; RWT, relative wall thickness.

**Table 3:** Multiple linear regression analysis of correlation between blood lipid, BMI, blood glucose, insulin, systolic blood pressure (SBP), body fat percentage, and parameters related to ventricular morphology.

Parameters	LVM		LVMI		RWT	
	*r*	*P* value	*r*	*P* value	*r*	*P* value
BMI	3.21	0.002	0.11	0.92	1.23	0.20
BFP	0.81	0.42	1.11	0.27	0.94	0.35
Systolic blood pressure (mmHg)	2.61	0.01	−0.46	0.65	2.50	0.013
Diastolic blood pressure(mmHg)	0.72	0.47	−1.2	0.23	−0.33	0.74
Fasting blood glucose	0.69	0.49	−0.16	0.88	0.64	0.52
Fasting insulin level	−0.15	0.89	0.62	0.54	−0.39	0.69
TG (mmol/L)	0.87	0.39	0.61	0.54	1.96	0.052
Total cholesterol (mmol/L)	0.17	0.86	0.63	0.53	0.29	0.78
HDL-C (mmol/L)	−1.56	0.12	−2.45	0.015	−2.35	0.02
TG/HDL ratio	−0.91	0.36	−0.37	0.71	−2.02	0.046

BMI, body mass index; BFP, body fat percentage; TG, triglycerides; HDL-C, high-density lipoprotein cholesterol; LVM, left ventricular myocardial mass; LVMI, left ventricular mass index; RWT, relative wall thickness.

### Comparison of body fat and blood lipid indicators among children with different left ventricular structural patterns

Based on the values of LVMI and RWT, obese children were categorized into four different left ventricular structural patterns. The differences in BFP and blood lipid profiles among these groups were compared and the results are presented in Table 4. One-way analysis of variance (ANOVA) revealed significant differences in BFP (F = 3.388, *P *= 0.02) and HDL-C (F = 2.917, *P *= 0.037) among the different left ventricular structural pattern groups. However, no significant differences were observed in other indicators.

**Table 4:** Levels and comparison of blood lipid profiles among obese children with four different left ventricular morphologies.

Ventricular morphology	Normal (*n* = 65)	Concentric remodeling (*n* = 12)	Concentric hypertrophy (*n* = 30)	Eccentric hypertrophy (*n* = 33)	F	*P* value
BMI	29.09 ± 4.80	28.43 ± 2.93	30.09 ± 4.70	30.02 ± 4.85	0.667	0.58
Body fat percentage (%)	40.48 ± 7.16	37.61 ± 5.96	43.03 ± 5.99	43.17 ± 4.92	3.388	0.02
Triglycerides (mmol/L)	1.49 ± 0.88	1.54 ± 0.75	1.73 ± 0.67	1.69 ± 0.78	0.820	0.485
Total cholesterol (mmol/L)	4.02 ± 0.78	3.87 ± 0.68	4.12 ± 0.74	4.26 ± 0.94	0.965	0.411
High-density lipoprotein (mmol/L)	1.21 ± 0.22	1.14 ± 0.31	1.09 ± 0.20	1.11 ± 0.18	2.917	0.037
TG/HDL ratio	1.35 ± 1.09	1.51 ± 1.06	1.61 ± 0.55	1.60 ± 0.90	0.77	0.52

Comparison of blood lipid profiles among different left ventricular structural pattern groups was conducted using one-way analysis of variance (ANOVA). Normal: Both relative wall thickness (RWT) and left ventricular mass index (LVMI) were within normal range. Concentric Remodeling: Increased RWT and normal LVMI. Concentric Hypertrophy: Increased RWT and LVMI. Eccentric Hypertrophy: Normal RWT and increased LVMI.

## Discussion

Childhood obesity has become an increasingly concerning health issue in recent years (11). The excessive accumulation of body fat can affect many organs and systems of children, with the cardiovascular system being the most vulnerable (12). The data of our study provides additional support that childhood obesity is independently associated with changes in cardiac structure and function. Previous studies on obese children have mostly utilized BMI as a screening criterion. However, BMI is merely a ratio of body weight to height and does not fully reflect the distribution of body fat (13). The essence of obesity lies in the excessive accumulation of body fat (14). Therefore, when assessing obesity-related risks, BMI has certain limitations and its assessment result may not accurately reflect the occurrence of obesity. On the other hand, the use of bioelectrical impedance analysis to measure BFP offers greater accuracy and convenience when combined with BMI, making it more suitable for screening larger populations (15). According to the criteria for diagnosing BFP, a BFP of ≥25% for boys and ≥30% for girls is defined as obesity. In this study, we utilized a combination of BMI and BFP (BF%) as a screening technique for obesity, which provided higher accuracy.

Based on our study results, we observed that children in the obese group had higher height, weight, BMI, BFP and blood pressure compared to the control group. Furthermore, echocardiographic examinations revealed that children in the obese group exhibited a significant increase in LVIDd, IVSd, LVPWd, LVM and RWT, while a decrease in the LVEF, confirming previous research findings (16). These findings indicate that obesity can elevate blood pressure and cardiac workload, as well as perhaps impair left ventricular function. Despite the absence of noticeable clinical symptoms in obese children, their cardiac structures are altered, and subclinical left ventricular dysfunction may be present. Failure to address this condition over a prolonged period can put additional strain on the heart, leading to myocardial hypertrophy, cardiac enlargement, increased myocardial oxygen demand, impaired left ventricular diastolic function, and ultimately, compromised overall cardiac function and heart failure (17).

LVH is widely considered as the primary indicator of cardiovascular target organ damage (10). Akin to primary hypertension, it is often asymptomatic in clinical practice. Research has revealed that the accumulation of fat within the left ventricle is a direct risk factor for LVH (18). In obese children, this is primarily manifested through abnormalities in lipid profiles (19). LVH is a frequent echocardiographic finding in obese children and cannot be solely explained by hemodynamic factors (20). Conversely, alterations in lipid levels, such as elevated TG, TC, and LDL-C, and reduced HDL-C, are common lipid metabolism irregularities that may contribute to myocardial hypertrophy in obese children (16, 21). In this study, we also found increased TG and TC as well as decreased HDL-C in the obese children. TG and TC are not soluble in water or blood, so the body wraps them into tiny protein-covered particles called lipoproteins. The lipoproteins most relevant to heart diseases are LDL and HDL, which act within the walls of blood vessels to promote and inhibit atherosclerosis, respectively (22, 23). Hyperlipidemia may induce left ventricular remodeling and LVH through various mechanisms such lipid accumulation within or around myocardial cells, local and systemic inflammation, insulin resistance, and oxidative stress (24).

Moreover, this study revealed a strong correlation between the lipid profiles of obese children and indicators of left ventricular remodeling, including LVM, LVMI, and RWT. RWT serves as a crucial indicator of ventricular remodeling and has been identified as an independent prognostic factor for patients with acute decompensated heart failure, irrespective of their ejection fraction status (25). A higher RWT value is associated with a poorer prognosis. Systolic blood pressure exhibited a negative correlation with HDL-C in obese individuals. HDL-C demonstrated a negative correlation with LVMI, and RWT, with the strongest association observed with LVMI. Additionally, a statistical correlation was found between the ratio of TG/HDL-C and LVMI. ANOVA demonstrated significant differences in HDL-C levels among children with different ventricular geometries, with the concentric hypertrophy group displaying the lowest HDL-C values. Conversely, no significant differences were observed in TC and TG levels among different groups.

HDL-C, commonly referred to as “good” cholesterol, is essential for countering the development of atherosclerosis (26). It facilitates the transportation of cholesterol from peripheral tissues to the liver for metabolism and subsequent excretion through bile. HDL-C exerts its anti-atherosclerotic effects through various mechanisms, including the promotion of reverse cholesterol transport (RCT), antioxide, and anti-inflammation, thereby safeguarding the cardiovascular system (27, 28). The incidence of coronary heart disease, atherosclerosis, and related cardiovascular disorders is highly inversely correlated with HDL-C levels, as consistently shown by epidemiological and clinical investigations (29). A decrease in HDL-C levels is associated with a higher risk of cardiovascular pathologies. A previous research conducted by Zhu Yuxing explored the association between lipid profiles, left ventricular structure and function, and chronic kidney disease (CKD) stages 4–5 in non-dialysis patients (30), and it revealed that the prevalence of LVH exceeded 70% in CKD patients and increased as renal function declined. Moreover, in comparison to the non-hypertrophy group, CKD patients with LVH exhibited much lower HDL-C levels, with a statistically significant difference. Concentric hypertrophy represents the cardiac remodeling pattern that exhibits the closest correlation with mortality (19, 31). This remodeling pattern is often accompanied by compromised cardiac systolic function and elevated blood pressure, which potentially enhances the risk of cardiovascular disease and mortality rates, specifically in obese children (32). Notably, our study indicated a substantial decrease in HDL-C levels in the concentric hypertrophy group as opposed to the other three pattern groups, thereby suggesting a significant association between reduced blood HDL-C levels and the occurrence of LVH in obese children and subsequently increased risk of cardiovascular disease.

There are some limitations in this study. Firstly, the sample size is relatively small, which may restrict the generalizability of the results. Secondly, the collection of cardiac ultrasound structural and functional indicators may not be comprehensive. In future research, a multicenter collaborative approach can be employed to increase the sample size, which will enhance the reliability and representativeness of the study. Additionally, further investigation into the changes in other systems among obese children is warranted, as this will contribute to a comprehensive understanding of the relationship between obesity and cardiovascular health.

## Conclusion

This study represents an initial investigation into the relationship between changes in left ventricular configuration and lipid profiles among obese children. The results demonstrate that obese children indeed experience alterations in left ventricular configuration, which show statistically significant correlations with changes in lipid profiles. Among the various lipid profile indicators examined, HDL-C stands out as a crucial serum factor for cardiovascular protection. It exhibits the closest association with left ventricular configuration indicators and displays significant differences among children with different ventricular configurations. Specifically, a lower HDL-C value indicates a higher risk of cardiovascular damage and increased severity of LVH.

## Data Availability

The original contributions presented in the study are included in the article/Supplementary Material, further inquiries can be directed to the corresponding author.
